# Evolution of Yeast Consortia during the Fermentation of Kalamata Natural Black Olives upon Two Initial Acidification Treatments

**DOI:** 10.3389/fmicb.2017.02673

**Published:** 2018-01-10

**Authors:** Stamatoula Bonatsou, Spiros Paramithiotis, Efstathios Z. Panagou

**Affiliations:** ^1^Laboratory of Microbiology and Biotechnology of Foods, Department of Food Science and Human Nutrition, Agricultural University of Athens, Athens, Greece; ^2^Laboratory of Food Quality Control and Hygiene, Department of Food Science and Human Nutrition, Agricultural University of Athens, Athens, Greece

**Keywords:** black olives, Kalamata variety, fermentation, molecular identification, yeasts

## Abstract

The objective of this study was to elucidate the yeast consortia structure and dynamics during Greek-style processing of Kalamata natural black olives in different brine solutions. Olives were subjected to spontaneous fermentation in 7% (w/v) NaCl brine solution (control treatment) or brine acidified with (a) 0.5% (v/v) vinegar, and (b) 0.1% (v/v) lactic acid at the onset of fermentation. Changes in microbial counts, pH, acidity, organic acids, sugars, and alcohols were analyzed for a period of 187 days. Yeast consortia diversity was evaluated at days 4, 34, 90, 140, and 187 of fermentation. A total of 260 isolates were characterized at sub-species level by rep-PCR genomic fingerprinting with the oligo-nucleotide primer (GTG)_5_. The characterization of yeast isolates at species level was performed by sequencing of the D1/D2 domain of 26S rRNA gene. Results showed that yeasts dominated the process presenting a relatively broad range of biodiversity composed of 11 genera and 21 species. No lactic acid bacteria (LAB) or *Enterobacteriaceae* could be enumerated after 20 and 10 days of fermentation, respectively. The dominant yeast species at the beginning were *Aureobasidium pullulans* for control and vinegar acidification treatments, and *Candida naeodendra* for lactic acid treatment. Between 34 and 140 days the dominant species were *Candida boidinii, Candida molendinolei* and *Saccharomyces cerevisiae*. In the end of fermentation the dominant species in all processes were *C. boidinii* and *C. molendinolei*, followed by *Pichia manshurica* and *S. cerevisiae* in lactic acid acidification treatment, *P. manshurica* in vinegar acidification treatment, and *Pichia membranifaciens* in control fermentation.

## Introduction

Table olives are a well-known fermented vegetable of the Western world with a great impact on the economy of the Mediterranean countries, which have an outstanding contribution in the global production of processed olives amounting to *ca.* 30%, with Spain being the leading producer followed by Greece and Italy (International Olive Council [IOC], 2017). A renewed interest has been shown in the last years for the functional properties of table olives (Bevilacqua et al., 2012a; Kailis and Kiritsakis, 2017), which in relation to optimal nutrition as indicated by the Mediterranean diet (Bach-Faig et al., 2017) resulted in increased consumption of table olives worldwide of *ca.* 2.5 million tons for the period 2015–2016 (International Olive Council [IOC], 2017). The Greek table olive sector has an important contribution in the economy of the country. According to the Interprofessional Association for Table Olives (DOEPEL, 2017), about 62,000 farmers and more than 100 companies are involved in the primary and secondary production of table olives. The annual production of processed table olives exceeds 200,000 tons, from which 85% is exported, representing 9.2% of the exports of Greek agricultural products.

Although all trade preparations of table olives are produced in the country, Greece has a long tradition in the production of natural black olives in brine, using olives from cv. Conservolea and Kalamata, which are two of the most economically important varieties for table olive processing in the country (Grounta et al., 2017). In the Greek processing system, olives are placed directly in brine solution with salt concentration of 8–10% (w/v) or even higher according to local practices, without any prior debittering pre-treatment. During the process, olives are subjected to spontaneous fermentation resulting in a characteristic final product with fruity aroma and a slightly bitter taste (Bleve et al., 2015). Diverse microbial populations are involved in olive fermentation including members of lactic acid bacteria (LAB) and yeasts which dominate the fermentation (Garrido-Fernández et al., 1997; Hurtado et al., 2008). These microorganisms through their metabolic activities in fermenting brines determine the organoleptic profile and the stability of the final product.

Taking into account that yeasts have a central role in the fermentation of natural black olives and the development of the final organoleptic characteristics, assessment of the biodiversity of yeast communities is fundamental for this trade preparation of table olives. Moreover, based on recent scientific findings, the contribution of yeasts in table olive processing has been reconsidered, giving emphasis on the beneficial aspects of yeasts due to their biotechnological and functional traits. For instance, yeasts have the ability to enhance the aroma and taste of fermented olives through the activity of specific enzymes such as lipases and esterases, that increase the free fatty acid content and thus result in the formation of several aromatic compounds such as ethanol, glycerol, higher alcohols and other desirable volatile compounds. Furthermore, biodegradation of polyphenols by specific yeast strains through the activity of β-glucosidase could lead to olive debittering without the use of any chemical treatment. In addition, the ability of specific yeast strains to produce enzymes such as phosphatases and phytases is an important technological feature as these enzymes can degrade phytic complexes and release inorganic phosphorous to the cells. Apart from the technological characteristics, yeasts exhibit probiotic potential such as tolerance through the gastrointestinal tract, inhibition of pathogens, adhesion to intestinal Caco-2 cell lines, and immune stimulation (Psani and Kotzekidou, 2006; Olstorpe et al., 2009; Arroyo-López et al., 2012b,c; Bevilacqua et al., 2012b; Tofalo et al., 2013; Bleve et al., 2015; Bonatsou et al., 2015, 2017b; Porru et al., 2018). Moreover, the ability of yeasts to co-aggregate on the surface of olives together with LAB and establish poly-microbial communities (biofilms) has been reported recently (Arroyo-López et al., 2012a; Grounta and Panagou, 2014; Benítez-Cabello et al., 2015) providing new perspectives for their use as multifunctional starters in Greek-style table olive processing.

In the past, identification of yeasts from Greek-style black olive fermentation has been reported (Kotzekidou, 1997) for industrially fermented olives, although no information was provided for the table olive varieties used. In another study (Nisiotou et al., 2010), the yeast succession and dominance in spontaneously fermented cv. Conservolea black olives in different brine solutions was reported at three different time points of fermentation. However, in the case of cv. Kalamata black olives the evolution of yeast microbiota during processing has been rarely explored. In a recent study (Bleve et al., 2015), molecular identification of LAB and yeast species with specific technological properties (i.e., presence of beta-glucosidase and inability for biogenic amine production) was reported during the spontaneous fermentation of cv. Conservolea and Kalamata natural black olives, in an attempt to develop a protocol for the pre-selection of fermentation starters. In another work (Tufariello et al., 2015), the dominance of inoculated yeast and LAB strains was reported for the same table olive varieties during Greek-style processing using molecular analyses. The aim of the present study was to assess the evolution of yeast community structure during the spontaneous fermentation of cv. Kalamata natural black olives upon different initial acidification treatments (with vinegar and lactic acid) by a multidisciplinary approach comprising chemical analyses and molecular profiling of yeast consortia.

## Materials and Methods

### Olive Samples and Fermentation Procedures

Kalamata natural black table olives (*Olea europea* var. *ceraticarpa*) were harvested in early December 2015 at the appropriate stage of ripeness to be processed according to the Greek-style method. The raw material was kindly provided by Konstantopoulos S.A. table olive industry located in Katerini, Northern Greece, subjected to quality control by the provider and transported within 24 h to the Agricultural University of Athens. Fermentation was undertaken in 12 total volume screw-capped plastic vessels, containing 8 kg of olives and 4 L of freshly prepared 7.0% (w/v) NaCl brine (control treatment) or brine acidified at the onset of fermentation with (a) 0.5% (v/v) vinegar (*ca.* 6%, v/v, acetic acid), and (b) 0.1% (v/v) lactic acid (90%, Sigma) at the same salt concentration. The different acidification treatments with vinegar or lactic acid at these concentrations were selected since they are increasingly employed by processors of Kalamata black olives in Greece today. All treatments were performed in duplicate and the fermentation vessels were maintained at room temperature for an overall period of 187 days (*ca.* 6 months). During the process, salt concentration was adjusted to the initial value of 7.0% by periodic additions of coarse salt in the brine.

### Microbiological Analyses

Olive samples were analyzed at 21 time points throughout fermentation (at days 1, 4, 7, 11, 15, 19, 22, 27, 34, 42, 50, 57, 66, 75, 90, 105, 118, 140, 153, 168, and 187) to determine the evolution of the indigenous microbiota on the surface of Kalamata olives. For this reason, four olives were randomly sampled at different depths from each fermentation vessel and the seed was removed using a sterile scalpel and forceps under aseptic conditions. Ten grams (10 g) of olive pulp were aseptically added in 90 mL sterile ¼ Ringer’s solution and homogenized in a stomacher (LabBlender, Seward Medical, London, United Kingdom) for 60 s at room temperature. The resulting suspension was serially diluted in the same diluent and 1 or 0.1 mL of the appropriate dilutions were mixed or spread on the following agar media to enumerate the main microbial groups driving fermentation, namely: (i) Lactic acid bacteria (LAB) on de Man-Rogosa-Sharpe medium (MRS; 401728, Biolife, Milan, Italy) adjusted to pH 5.7 and supplemented with 0.05% (w/v) cycloheximide (AppliChem GmbH, Darmstadt, Germany), overlaid with the same medium and incubated at 25°C for 72 h; (ii) Yeasts and Molds on Rose Bengal Chloramphenicol agar (RBC; supplemented with selective supplement X009, Bury, United Kingdom), incubated at 25°C for 48 h; and (iii) *Enterobacteriaceae* on Violet Red Bile Glucose agar (VRBGA; Biolife, Milan, Italy), incubated at 37°C for 24 h. All plates were examined visually for typical colony types and morphological characteristics that were associated with each growth medium. Moreover, the selectivity of each medium was routinely checked by microscopic examination of smears prepared from randomly selected colonies obtained from the media and Gram staining. Results were expressed as log values of colony forming units per gram (log CFU/g) of olives.

### Physicochemical Analyses

Physicochemical analyses were undertaken throughout the fermentation in the brines to monitor the changes of pH, titratable acidity, salt concentration, organic acids (lactic, acetic, malic, citric, tartaric, succinic), sugars (glucose, fructose), and alcohols (ethanol, glycerol). Specifically, pH values were monitored using a digital pH-meter (Russel Inc., Boston, MA, United States). Titratable acidity and salt concentration were determined according to Garrido-Fernández et al. (1997). Finally, organic acids, sugars and alcohols were analyzed by HPLC based on the protocol described by Bleve et al. (2015). All analyses were undertaken in duplicate and results are expressed as mean values ± standard deviation.

### Yeast Isolation and Characterization

Yeast colonies were selected from the RBC plates according to Harrigan and McCance (1976) and purified by successive streaking on the same medium. Pure cultures were maintained at -80°C in Yeast Mold (YM) medium supplemented with 20% glycerol. Yeast species diversity was evaluated at five different sampling times, namely 4 (T_4_), 34 (T_34_), 90 (T_90_), 140 (T_140_), and 187 (T_187_) days of fermentation in order to describe the evolution of yeast consortia at the beginning, middle and end of fermentation together with two additional intermediate points during the course of fermentation. A total of 271 isolates (i.e., 15–20 colonies per plate, sampling time and fermentation process) were subjected to sequencing of the D1/D2 domain of 26S rRNA gene for species assignment and microsatellite-primed PCR with the oligo-nucleotide primer (GTG)_5_ for their characterization at sub-species level.

DNA extraction was performed according to Querol et al. (1992) modified by using lyticase (2.5 U/mL) (Lyticase from *Arthrobacter luteus*, Sigma–Aldrich, Germany) for yeast cell lysis. Amplification and sequencing of the D1/D2 domain of 26S rRNA gene was performed according to Pateraki et al. (2014). The taxonomic affiliation was assessed using the BLAST software in the GENBANK collection. Genotypic diversity at sub-species level was assessed in a final volume of 25 μl containing 3 mM MgCl_2_, 2 mM (GTG)_5_ primer, 2 U Taq polymerase (KAPA Taq PCR kit, KAPA Biosystems, United States), 0.2 mM dNTP’s (Invitrogen) and 75 ng of template DNA. Amplification was carried out in a thermocycler (Applied Biosystems, Bedford, MA, United States) under the following conditions: initial denaturation at 94°C for 5 min; 30 cycles of 94°C for 30 s, 40°C for 1 min, and 72°C for 8 min; and a final extension at 72°C for 16 min. All PCR products were analyzed by electrophoresis in 1.5% agarose at 100 V for 1.5 h. Gels were scanned with the GelDoc system (Bio-Rad, Hercules, CA, United States). Conversion, normalization, and further analysis were performed using the Dice coefficient and the unweighted pair group method with arithmetic mean (UPGMA) cluster analysis with Bionumerics software version 6.1 (Applied Maths, Sint-Martens-Latem, Belgium). Results were expressed as isolation frequency (%) that was determined as the number of isolates of a particular yeast species divided by the total number of yeast isolates at a given sampling time.

### Statistical Analysis

The data were subjected to one-way analysis of variance using Statistica 7.1 software (Statsoft Inc., Tulsa, OK, United States) to check for significant differences among microbiological and physicochemical characteristics according to the different fermentation treatments. Differences between means were determined by the statistical LSD test at *p* ≤ 0.05.

## Results and Discussion

### Population Dynamics and Physicochemical Changes

**FIGURE 1:**
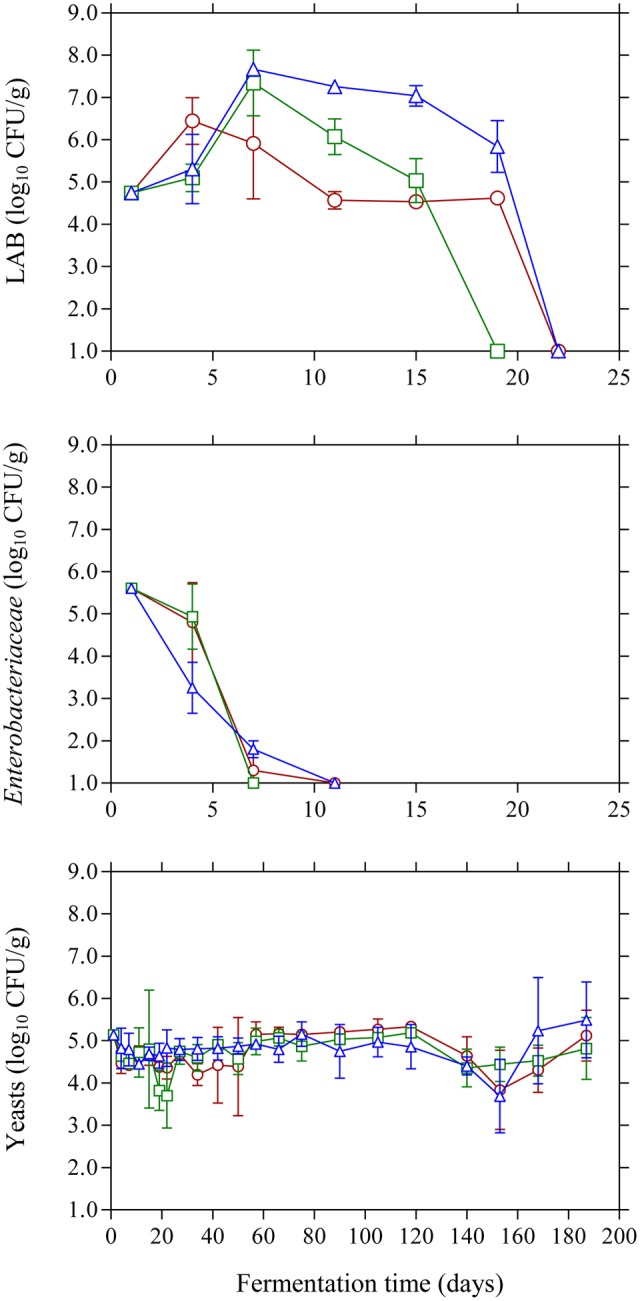
Changes in the population of LAB, *Enterobacteriaceae*, and yeasts on olive drupes during the spontaneous fermentation of Kalamata natural black olives in brines with different initial acidification treatments. (○) without brine acidification (control); (□) brine acidified with 0.5 % (v/v) vinegar; (△) brine acidified with 0.1% (v/v) lactic acid. Data points are mean values of duplicate fermentations ± standard deviation.

The population dynamics of the main microbial groups on olive drupes during the different fermentation processes is presented in **Figure 1**. Specifically, at the beginning of the process LAB population was 4.7 log_10_ CFU/g and began to actively grow until day 7 where the highest counts (*p* ≤ 0.05) were observed in the acidified brines compared to control treatment. From this point, a progressive reduction in LAB population was observed until day 20 where no LAB could be detected on olive drupes. It needs to be noted that acidified brines favored higher growth profiles for LAB and this was more evident in the case of lactic acid where higher population (*p* ≤ 0.05) of *ca.* 6.0 log_10_ CFU/g was observed at day 20 compared with the other two treatments, possibly due to the acid-tolerant features of this bacteria group. The changes in the population of *Enterobacteriaceae* were very similar (no statistical differences) between acidified and control treatments. Specifically, they were detected at *ca.* 5.6 log_10_ CFU/g but decreased rapidly and were below the enumeration limit (1.0 log_10_ CFU/g) after 11 days of fermentation. Regarding the effect of the different initial acidification treatments, the use of vinegar reduced the survival time by 4 days compared to control and lactic acid acidification. The survival period of this microbial group should be reduced to a minimum as prolonged persistence may result in spoilage of Kalamata olives in the form of gas pockets and fissures on the drupes (Sánchez-Gómez et al., 2006). Finally, yeasts coexisted with LAB for the first 15–20 days and their population was maintained 2–3 log cycles below LAB counts. However, from this time point onward, yeasts became the dominant microbial group that controlled fermentation. Their growth pattern was similar in all treatments (no statistical differences) regardless of initial acidification of the brine presenting an average initial population of 5.2 log_10_ CFU/g that was maintained around this value throughout the process.

**FIGURE 2:**
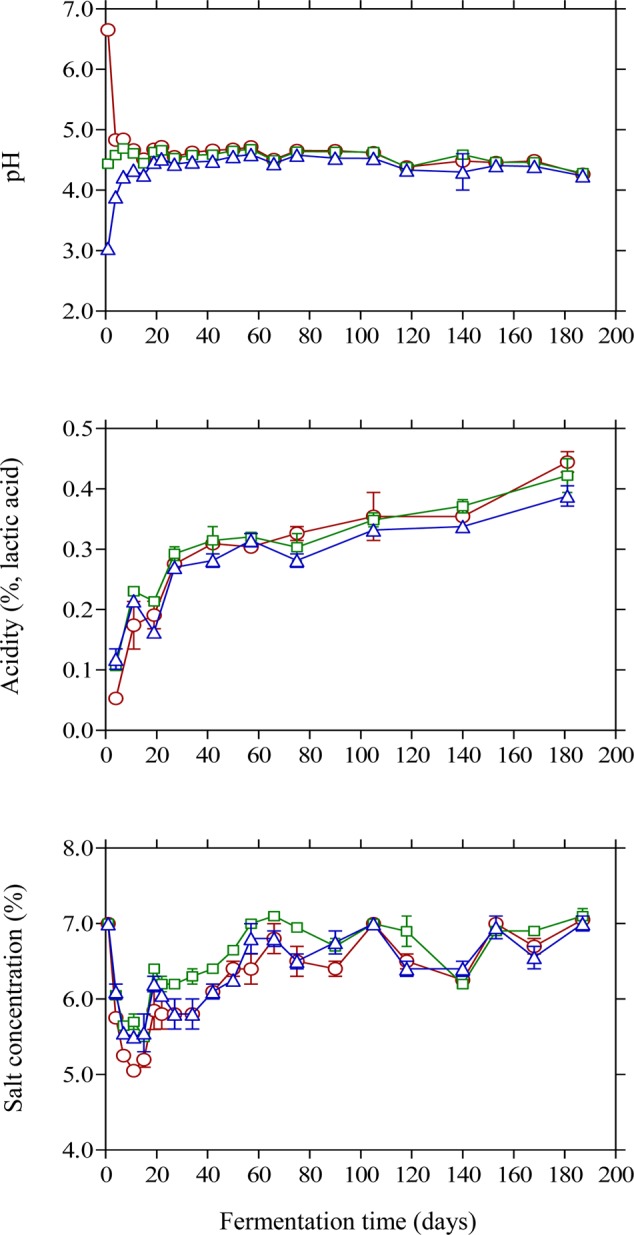
Changes in pH, titratable acidity and salt concentration in the brines of Kalamata natural black olives during spontaneous fermentation with different initial acidification treatments. (○) without brine acidification (control); (□) brine acidified with 0.5% (v/v) vinegar; (△) brine acidified with 0.1% (v/v) lactic acid. Data points are mean values of duplicate fermentations ± standard deviation.

The changes in pH and acidity were typical for Kalamata natural black olive fermentation. As expected, brine acidification affected the initial pH values (**Figure 2**). Thus, lactic acid acidified brine presented the lowest initial pH (3.04) followed by vinegar acidification (4.44) and finally the control (6.65) where no brine acidification was performed. In the case of brines acidified with lactic acid, pH presented a gradual increase until day 20 reaching a plateau at 4.52, whereas less variation in pH was observed for the vinegar acidification treatment. The final pH measured in the brines at the end of fermentation was similar in all treatments reaching a minimum value of 4.2. The effect of initial acidification was also evident in the initial values of titratable acidity in the brines, with control treatment presenting lower acidity values (0.05%) compared to lactic acid (0.12%) and vinegar (0.10%) treatments (**Figure 2**). However, the profile of acidity was similar in all cases presenting a gradual increase until the end of fermentation with final values of 0.38–0.44%. The obtained final values for pH and acidity differ from those reported in a previous work on Kalamata natural black olives (Bonatsou et al., 2017a), where the process was dominated by LAB instead of yeasts resulting in a vigorous lactic acid process with lower pH and higher acidity values. However, it must be underlined that the population dynamics of LAB is not fully comparable since in the latter work olives had been subjected to osmotic pre-treatment prior to processing and the brines had been supplemented with monosodium glutamate. In another study (Bleve et al., 2015) Conservolea and Kalamata olives were fermented naturally and the minimum pH values attained at the end of fermentation were 4.2–4.3 due to the dominance of yeasts over LAB, which is in good agreement with the final pH values reported in this work. It needs to be noted that despite the fact that yeasts were the dominant microbial group, the final values for pH and acidity are within the limits of the trade standard applying to table olives of the International Olive Council (International Olive Council [IOC], 2004) where for natural fermentations the maximum limit for pH and minimum acidity should be 4.3 and 0.3 %, respectively. Salinity in the brines was monitored throughout fermentation and adjusted to the initial value of 7% by periodic dry salt additions in the brines. Salt equilibrium was reached in *ca.* 2 months and until the end of the process salt concentration was maintained between 6 and 7%. It is characteristic that within the first 10 days of fermentation the salt level was reduced by 1.5–2.0% and reached concentrations as low as 5% in control treatment (**Figure 2**) favoring thus the growth of LAB that became the dominant microbial group at the early stage of fermentation. It must be underlined that the maximum population of LAB was observed within this time period where salt concentration reached its lowest levels in the brines. The continuous salt addition in the brines from day 10 onward to reach the desired level of 7% could have possibly resulted in a potential stress on LAB that started to decline rapidly and became undetectable after 20 days of fermentation.

**FIGURE 3:**
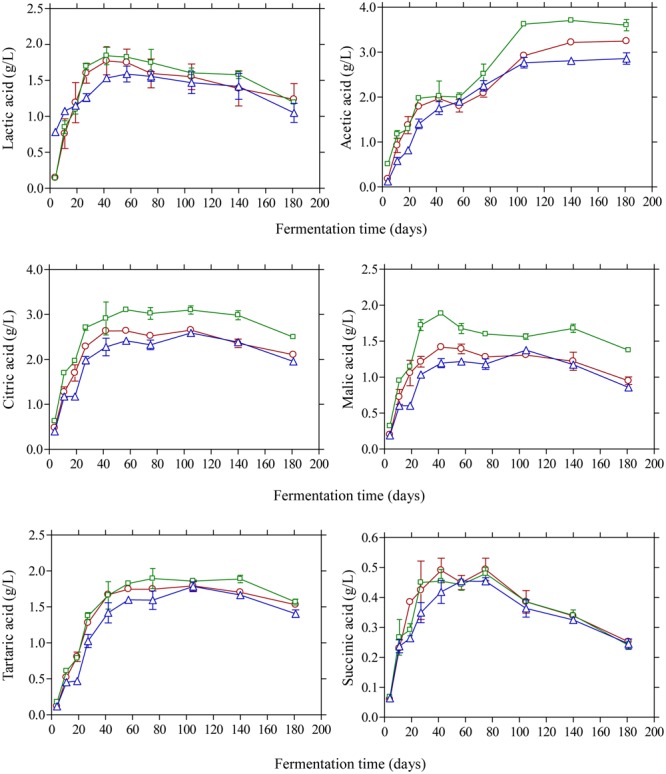
Changes in the concentration (g/L) of organic acids (lactic, acetic, citric, malic, tartaric, and succinic) during the spontaneous fermentation of Kalamata natural black olives in brines acidified with 0.5% (v/v) vinegar (□), 0.1% (v/v) lactic acid (△), and without brine acidification (○). Data points are mean values of duplicate fermentations ± standard deviation.

The changes in the concentration of organic acids in the brines are shown in **Figure 3**. Acetic acid was the main acid with considerable presence in the brines as detected by HPLC. Its concentration presented a gradual increase until day 105 and then remained unchanged until the end of the process. As expected, brines acidified with vinegar presented higher concentrations (*p* ≤ 0.05) at the end of fermentation (3.6 g/L) compared with control (3.2 g/L) and lactic acid acidified brines (2.6 g/L). The presence of acetic acid could be attributed to yeast activity (Querol and Fleet, 2006; Bleve et al., 2014) although the contribution of LAB in acetic acid production could also be taken into consideration, especially in the early stage of fermentation (until day 20), due to the potential of homo- and heterofermentative LAB to generate acetic acid from fermentable material under particular conditions of environmental stress as well as from the metabolism of citrate (Bobillo and Marshall, 1991; Laëtitia et al., 2014). Lactic acid was also detected in the brines in concentrations not exceeding 2.0 g/L throughout the process. It presented a gradual increase until day 40 followed by a steady decline thereafter without statistically significant differences among the treatments. As LAB were practically absent from the process, the concentration of this organic acid was lower compared with previously published works on Kalamata olives fermentation (Bleve et al., 2015; Tufariello et al., 2015; Bonatsou et al., 2017a). Citric, malic and tartaric acids were also detected in the brines presenting a similar pattern with concentrations not exceeding 3.0 and 2.0 g/L, respectively, whereas succinic acid was presented in lower concentrations (<0.5 g/L) without statistical differences at the end of fermentations.

**FIGURE 4:**
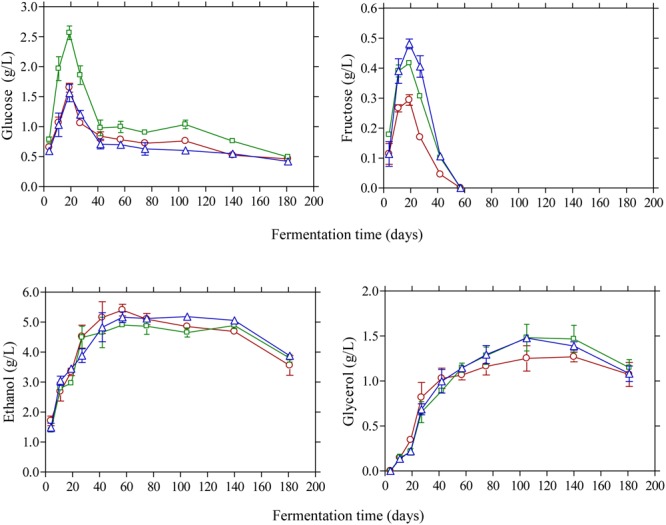
Changes in the concentration of glucose, fructose, ethanol, and glycerol (g/L) in the brines during processing of Kalamata natural black olives with different initial acidification treatments. (□) Brine acidified with 0.5 % (v/v) vinegar; (△) brine acidified with 0.1 % (v/v) lactic acid; (○) without brine acidification (control).

Glucose and fructose were the main sugars in the brines detected by HPLC (**Figure 4**). Glucose content rapidly increased at the early stage of fermentation and reached a maximum at day 20 and then decreased thereafter since it was consumed for microbial growth. Higher amounts of glucose were detected in the vinegar acidification treatment at this time compared with the other two fermentation procedures. This could be attributed to the fact that no LAB could be enumerated on olives at day 20, compared with the control and lactic acid acidification treatment where the counts of LAB were 4.6 and 5.8 log_10_ CFU/g, respectively (**Figure 1**). Thus, the different levels of glucose at day 20 reflect the different metabolic activity of bacteria in the brines at this particular time point. It is characteristic that at the end of fermentation glucose was not totally depleted but there was a remaining amount of *ca.* 0.5 g/L in the brines. A similar pattern was observed for fructose that was detected at lower levels in the brines compared with glucose. The concentration of this sugar in the brines never exceeded 0.5 g/L and it was undetectable after 60 days of fermentation. It has been reported that the prevalence of residual glucose over fructose could be attributed to the presence of some fructophilic non-*Saccharomyces* yeasts commonly found in the brine together with varietal characteristics of the fermented olives (Tufariello et al., 2015). Ethanol is a major metabolite which is produced by yeast fermentation which is important for the sensory properties of natural black olives (Fleming et al., 1969). Its concentration increased gradually until day 60 of fermentation reaching values 4.9–5.1 g/L followed by a gradual decrease thereafter until the end of the process where ethanol concentration was maintained at *ca.* 3.5–3.8 g/L. No significant differences among the fermentation profiles could be established for ethanol concentration, indicating that yeast growth was not affected by the different acidification treatments in the brines. Glycerol is an important secondary product of yeast metabolism of sugars (Erasmus et al., 2004; Swiegers et al., 2005). It is also important to yeasts because it protects the cell from osmotic stress since it is an effective compatible solute (Dickinson and Kruckeberg, 2006). Its concentration increased gradually until the 60th day of fermentation in levels not exceeding 1.5 g/L followed by a decrease afterward reaching final values of *ca.* 1.0 g/L. The presence of this compound in Kalamata natural black olives has been reported previously by Italian researchers (Bleve et al., 2015; Tufariello et al., 2015) in amounts comparable with those reported in this work. The production of ethanol and glycerol due to yeast activity together with other volatile compounds has important contribution in flavor development and texture maintenance during table olive processing (Arroyo-López et al., 2008, 2012b,c).

### Yeast Species Identification and Heterogeneity

**FIGURE 5:**
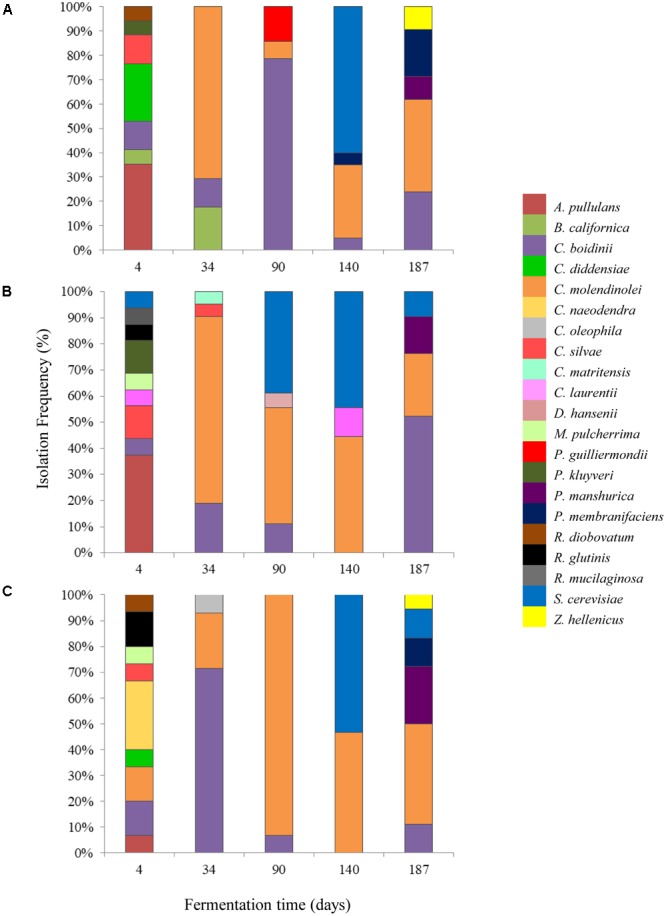
Isolation frequency (%) of yeasts during the spontaneous fermentation of Kalamata natural black olives in brines with different initial acidification treatments. **(A)** Without brine acidification (control); **(B)** brine acidified with 0.5% (v/v) vinegar; **(C)** brine acidified with 0.1% (v/v) lactic acid.

A total of 260 isolates were subjected to sequencing of the D1/D2 region of 26S-rRNA gene for their taxonomic assignment at species level as well as to (GTG)_5_ rep-PCR fingerprinting for their differentiation at subspecies level. The majority of the isolates were identified as *Candida molendinolei, Candida boidinii*, and *Saccharomyces cerevisiae*; another 18 species, namely *Aureobasidium pullulans, Barnettozyma californica, C. diddensiae, C. naeodendra, C. oleophila, C. silvae, Citeromyces matritensis, Cryptococcus laurentii, Debaryomyces hansenii, Metchnikowia pulcherrima, Pichia guilliermondii, Pichia kluyveri, P. manshurica, P. membranifaciens, Rhodotorula diobovatum, Rhodotorula glutinis, Rhodotorula mucilaginosa*, and *Zygoascus hellenicus* were also detected throughout fermentation. The yeast microecosystem composition during the three fermentation procedures assayed in this study is presented in **Figures 5A–C**. *C. molendinolei* and *C. boidinii* seemed to form a relatively stable dual species consortium since they were both detected from the 34th day of fermentation regarding the non-acidified brine and from the 4th day of fermentation regarding the acidified ones. A plethora of yeast species, namely *A. pullulans, B. californica, C. diddensiae, C. naeodendra, C. oleophila, C. silvae, C. matritensis, C. laurentii, C. bisporidii, D. hansenii, M. pulcherrima, P. guilliermondii, P. kluyveri, R. diobovatum, R. glutinis* and *R. mucilaginosa* was associated with the early stages of fermentation (until the 90th day) while significantly less, namely *P. manshurica, P. membranifaciens, S. cerevisiae* and *Z. hellenicus* in the final stages. This observation is consistent with a previous study undertaken on cv. Conservolea natural black olives to elucidate yeast diversity in different brine solutions (Nisiotou et al., 2010), in which a broader range of yeast species was also revealed at the beginning compared to the end of fermentation. This could be attributed to the fact that the species recovered at the beginning of fermentation originated from the initial microbiota adhered to the surface of olives and could be different to those present at the industrial environment where the resident yeast microbiota (e.g., in fermentation vessels) has an important contribution in fermentation (Botta and Cocolin, 2012).

The initial acidification of the brine with vinegar or lactic acid affected the heterogeneity of yeasts during the process. Thus, the dominant yeast species at the early stage of fermentation (T_4_) were *A. pullulans* for control and vinegar acidification treatments, with 35.3 and 37.5% isolation frequency, respectively, and *C. naeodendra* for lactic acid treatment (26.7%). *A. pullulans* is an ubiquitous oxidative yeast like species encountered on food surfaces and the phyllosphere (Zalar et al., 2008), which is inhibited after the first fermentation days (Sabate et al., 2002). Its presence at the initial stage of fermentation has been reported recently in green (Valenčič et al., 2010; Alves et al., 2012) and black olives (Nisiotou et al., 2010). *C. naeodendra* has been described as a new species of the *C. diddensiae* group (van der Walt et al., 1973) and it is differentiated from most members of this group on the basis of its marked lipolytic activity. Its presence on olives is reported for the first time in this work. In the middle stage of fermentation (T_90_) the dominant species were *C. boidini* in control fermentation (78.5% isolation frequency) and *C. molendinolei* in lactic acid and vinegar acidified brines with 93.3 and 44.4% isolation frequencies, respectively. *C. boidini* is very common in all table olives preparations (Nisiotou et al., 2010; Bautista-Gallego et al., 2011; Tofalo et al., 2013; Pereira et al., 2015; Porru et al., 2018), whereas *C. molendinolei* has been initially isolated from olive oil (Mari et al., 2016) and only recently from table olives (Mateus et al., 2016; Porru et al., 2018). Finally, at the end of fermentation (T_187_), apart from the above mentioned yeast species, *P. membranifaciens* was also recovered from control fermentation (19.0%) as well as *P. manshurica* from lactic acid (22.2%) and vinegar (14.3%) acidified brines. Both yeasts have been widely identified in table olives (Hurtado et al., 2008; Nisiotou et al., 2010; Bleve et al., 2014; Pereira et al., 2015), whereas *P. membranifaciens* may also influence fermentation by affecting yeast association through the production of killer toxins (Psani and Kotzekidou, 2006; Arroyo-López et al., 2008).

**FIGURE 6:**
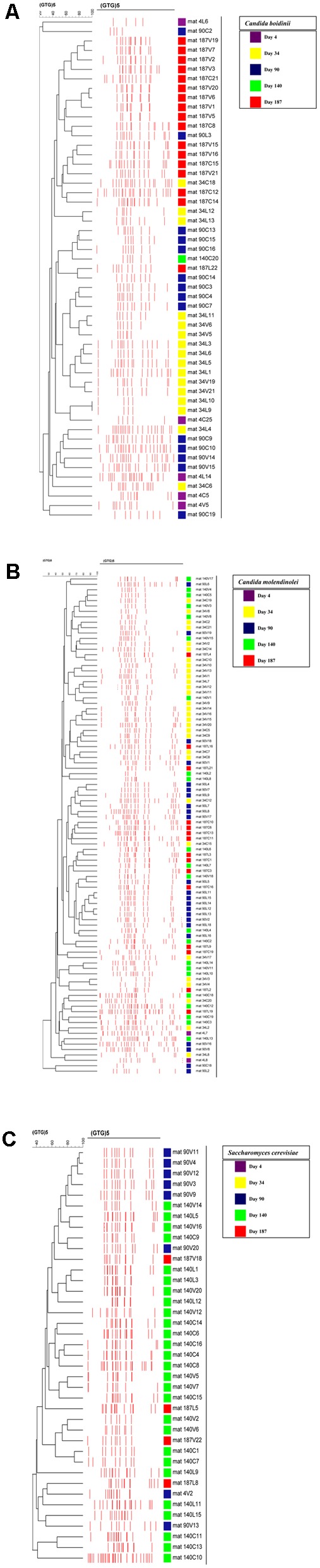
Cluster analysis of rep-PCR patterns of the dominant yeast species obtained during the spontaneous fermentation of Kalamata natural black olives: **(A)** *Candida boidinii*, **(B)** *Candida molendinolei*, **(C)** *Saccharomyces cerevisiae*. Distance is indicated by the mean correlation coefficient [*r* (%)] and clustering was performed by UPGMA analysis.

The cluster analysis of rep-PCR patterns of the yeast isolates obtained during fermentation without acidification (control) or with the addition of vinegar or lactic acid, respectively, is exhibited in Supplementary Figures 1a–c. Moreover, cluster analysis of each of the main yeast species identified (i.e., *C. molendinolei, C. boidinii*, and *S. cerevisiae*) was created and presented in **Figures 6A–C**. In all cases, with two exceptions different genotypic profiles were obtained from isolates belonging to the same species, which indicates effective differentiation at subspecies level. The exceptions refer to *S. cerevisiae* strains 90V_11_ and 90V_12_, as well as *C. boidinii* strains 34L_9_ and 34L_10_, that were isolated from the vinegar acidification treatment at day 90 and lactic acid treatment at day 34, respectively. The ability of (GTG)_5_ fingerprinting to differentiate yeasts at subspecies level has been adequately exhibited (Pateraki et al., 2014; Ramirez-Castrillon et al., 2014; Capece et al., 2016; Verspohl et al., 2017). Assessment of yeast population dynamics with this approach often reveals a succession at subspecies level, which is quite reasonable considering the spontaneous nature of fermentation.

## Conclusion

This work described for the first time the evolution of yeast consortia at species and sub-species level associated with cv. Kalamata natural black olive fermentation under different initial acidification procedures commonly employed by Greek processors. Despite the fact that LAB dominated at the early stage of fermentation, they could not eventually survive in the brines and fermentation was undertaken by yeasts that became the dominant microorganisms, presenting a broad range of biodiversity including 11 genera and 21 species. The different acidification agents (vinegar and lactic acid) employed in the fermentation affected the final composition of yeast species on olives. Given the important contribution of yeasts in natural black olive fermentation, the results of this work could provide information on yeast heterogeneity for one of the most economically important Greek table olive varieties. In addition, table olive research has been focused on the beneficial effects of yeasts during olive processing due to their biotechnological and probiotic potential. Thus, further work is underway to elucidate the multifunctional features of selected yeast species from this consortium to be used as starter cultures in inoculated fermentations of Kalamata black olives to provide new perspectives for this particular trade preparation of table olives.

## Author Contributions

SB performed the experiments, analyzed the data, and contributed in the preparation of the paper. SP was involved in the molecular characterization of yeast species. EP was involved in the experimental design, interpretation of the results, and revised the paper.

## Conflict of Interest Statement

The authors declare that the research was conducted in the absence of any commercial or financial relationships that could be construed as a potential conflict of interest.
