# Supplementary material for: Evolution of Yeast Consortia during the Fermentation of Kalamata Natural Black Olives upon Two Initial Acidification Treatments
Source: Front Microbiol. 2018 Jan 10;8:2673. doi: 10.3389/fmicb.2017.02673 (PMC5767579; doi:10.3389/fmicb.2017.02673)
Supplement: Supplementary file 1 [file Data_Sheet_1.docx]

**Supplementary Figure 1**. Cluster analysis of rep-PCR patterns of the dominant yeast species obtained during the spontaneous fermentation of Kalamata natural black olives in brines without initial acidification (a), acidified with 0.5% (v/v) vinegar (b), and 0.1% (v/v) lactic acid (c). Distance is indicated by the mean correlation coefficient [*r* (%)] and clustering was performed by UPGMA analysis.

Fig. 1a

Fig. 1b

Fig. 1c
